# Supplementary material for: Analysis of Pesticide Residues on Fruit Using Swab Spray Ionization Mass Spectrometry
Source: Molecules. 2023 Sep 14;28(18):6611. doi: 10.3390/molecules28186611 (PMC10537605; doi:10.3390/molecules28186611)
Supplement: Supplementary file 1 [file molecules-28-06611-s001.zip › molecules-2579050-supplementary.pdf]

Supplementary data

S1 Detection of pesticides on clementine, orange, and tomato

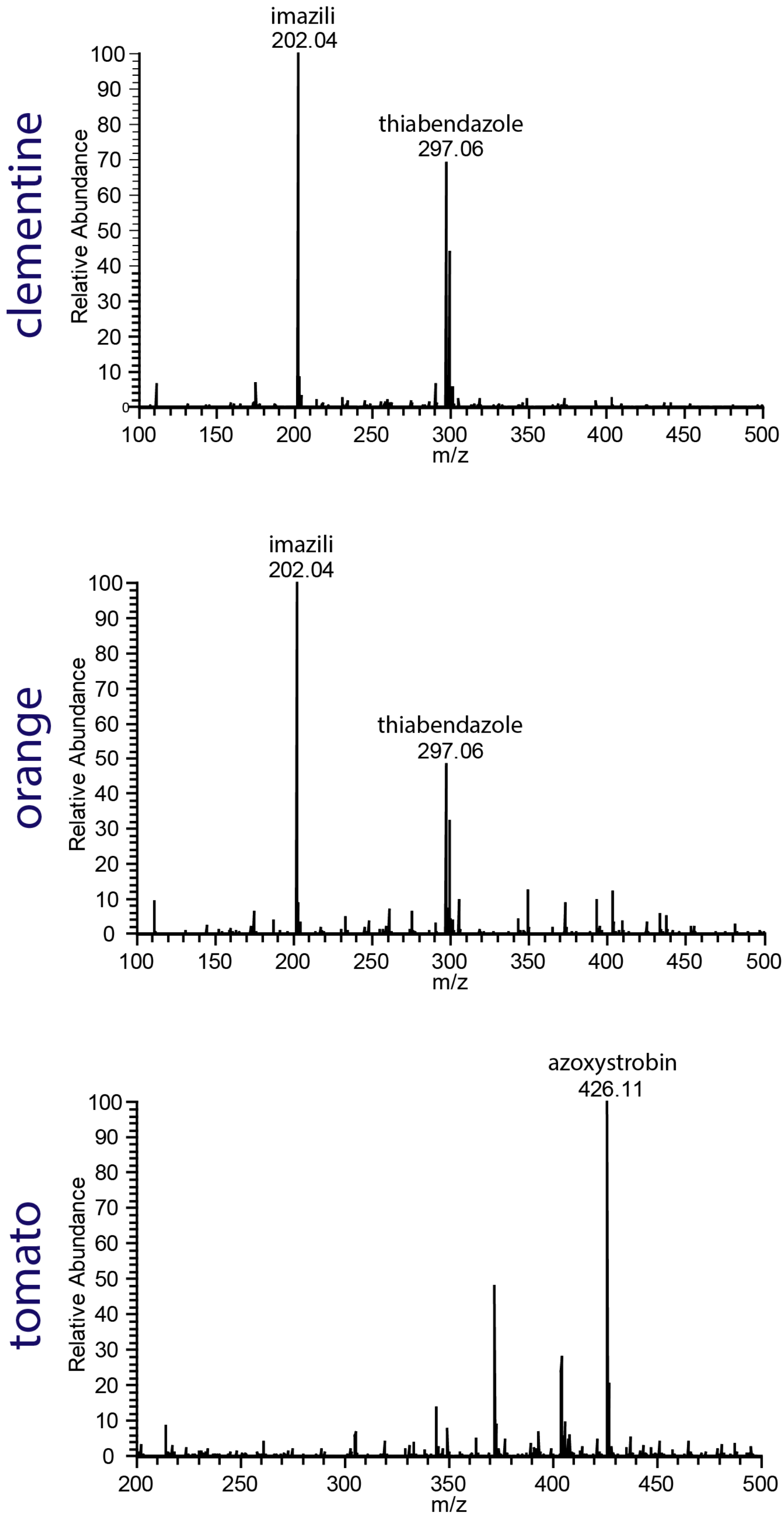

Figure S1 Full scan spectra illustrating the detection of several pesticides on fruit.

S2 Reproducibility

Table S1 Reproducibility of swab spray ionization mass spectrometry.

100 ng imazalil deposited directly on the swab head
(4 μl of 25 μg/ml imazalil solution in methanol)

**measurement**	**area**	**relative deviation from average value**
1	1.44E+09	1.91%
2	1.21E+09	-13.86%
3	1.21E+09	-14.44%
4	1.70E+09	20.92%
5	1.49E+09	5.47%
**standard deviation**	1.86E+08	
**average value**	1.41E+09	

S3 Depletion curves obtained by swab spray ionization analysis of boscalid depots

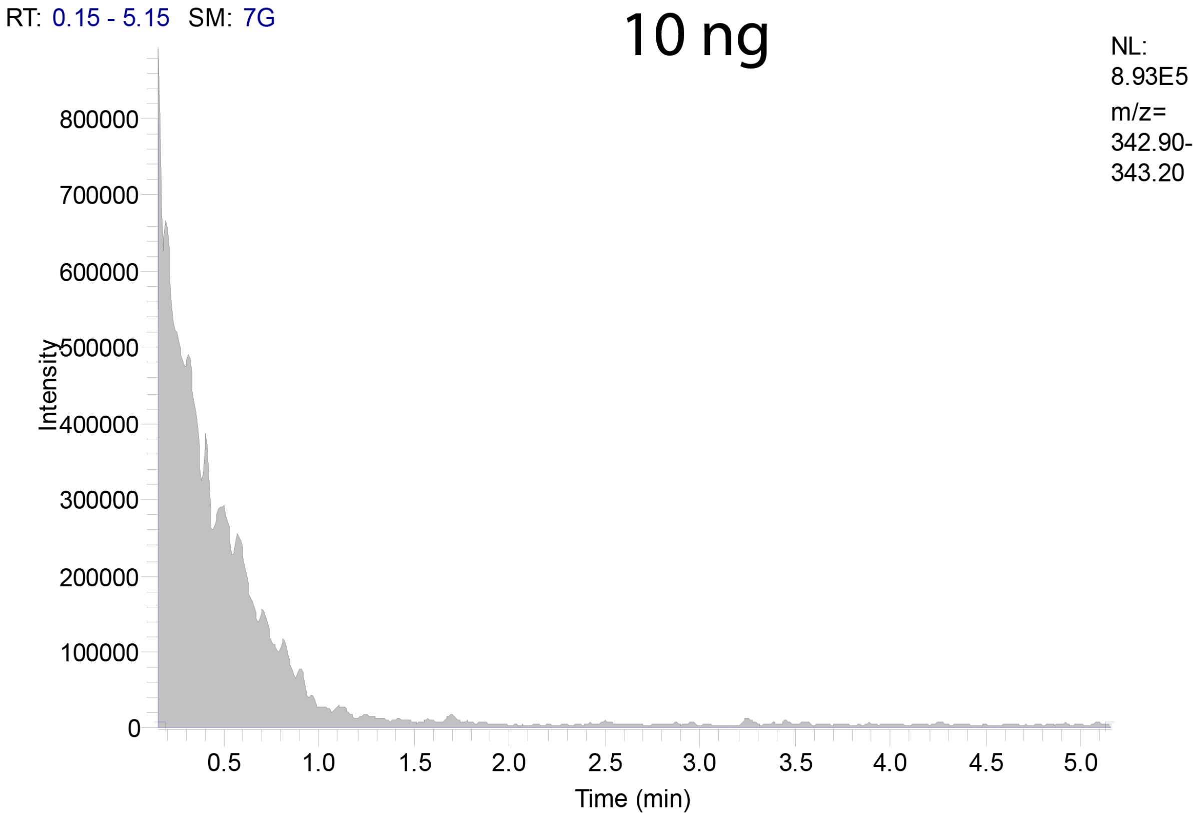

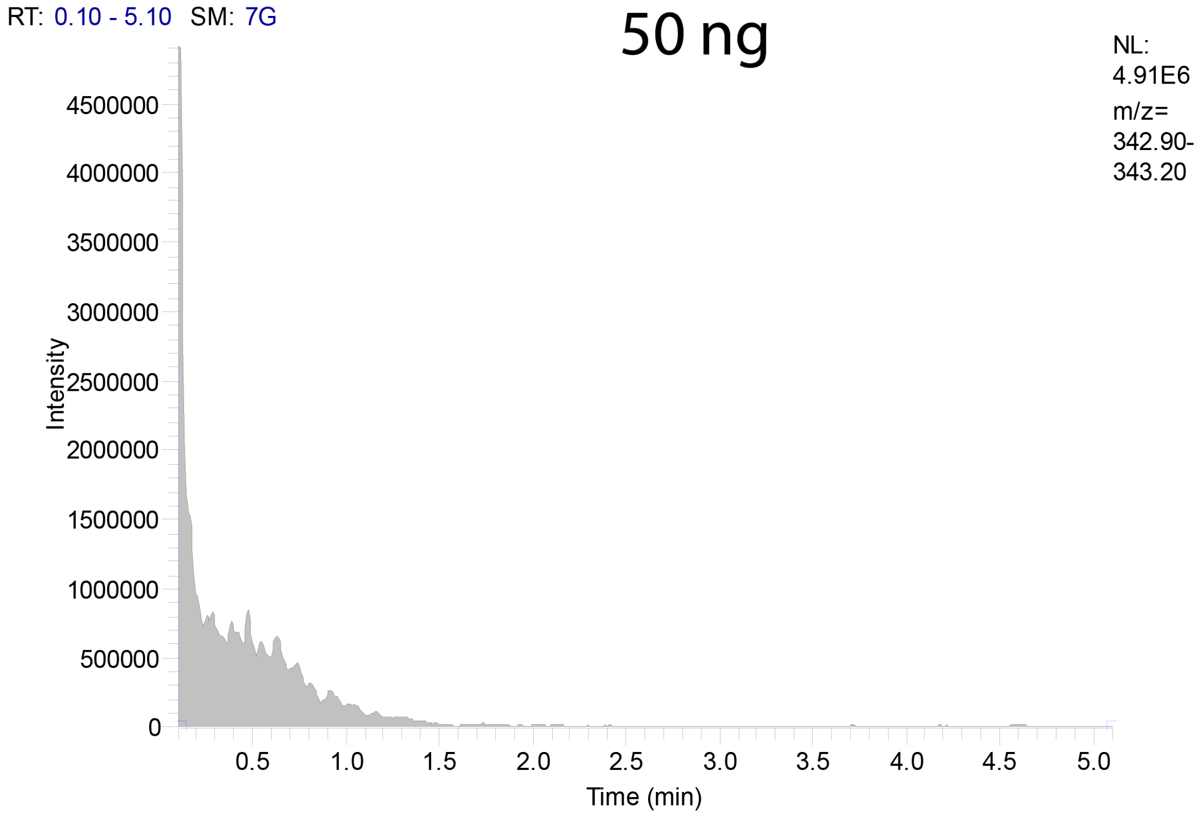

Figure S2 Depletion curves of 10 ng and 50 ng boscalid, obtained by an extracted ion current of m/z 342.90-343.20 (gaussian smoothing 7 points).

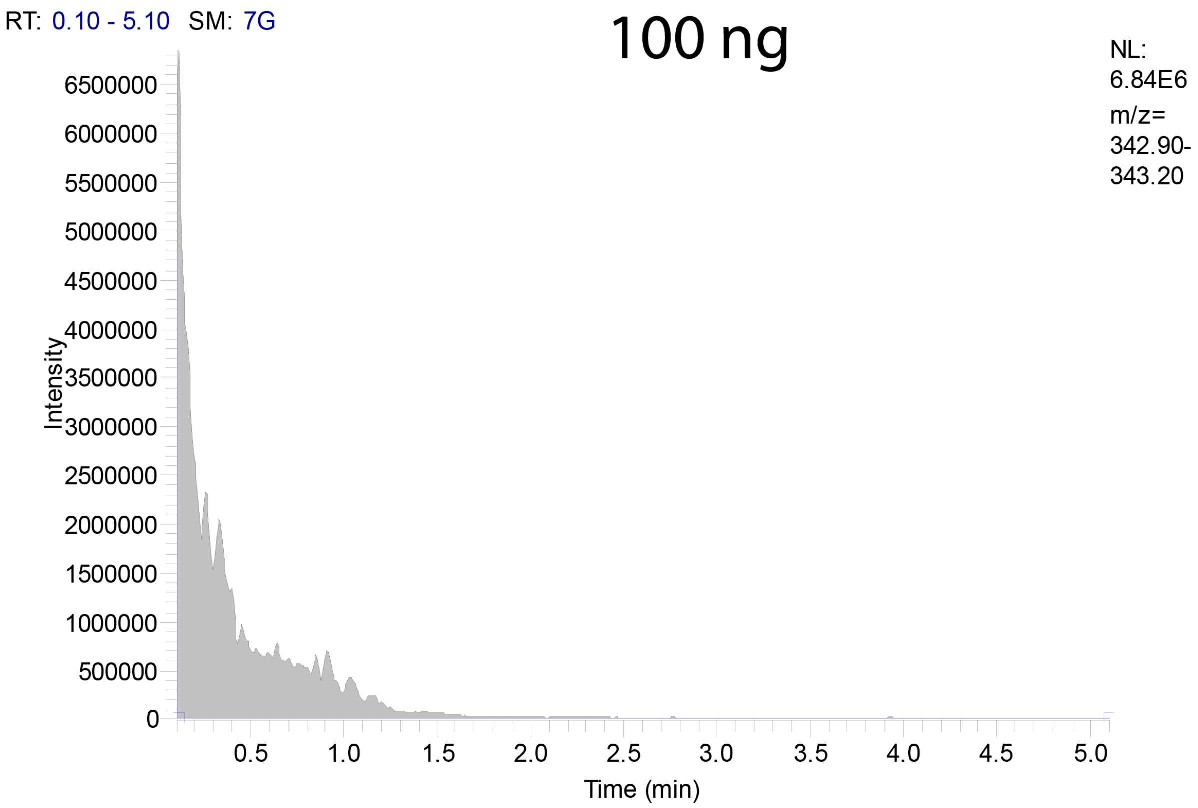

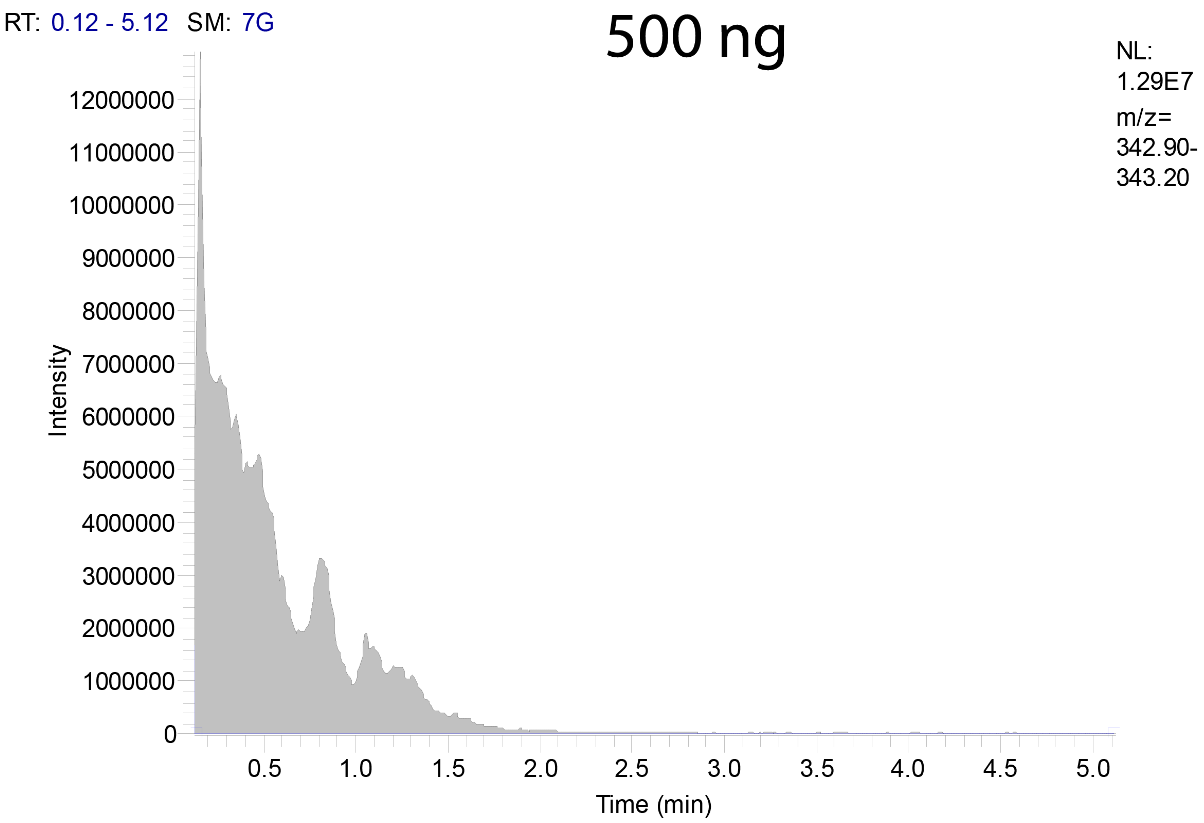

Figure S3 Depletion curves of 100 ng and 500 ng boscalid, obtained by an extracted ion current of m/z 342.90-343.20 (gaussian smoothing 7 points).

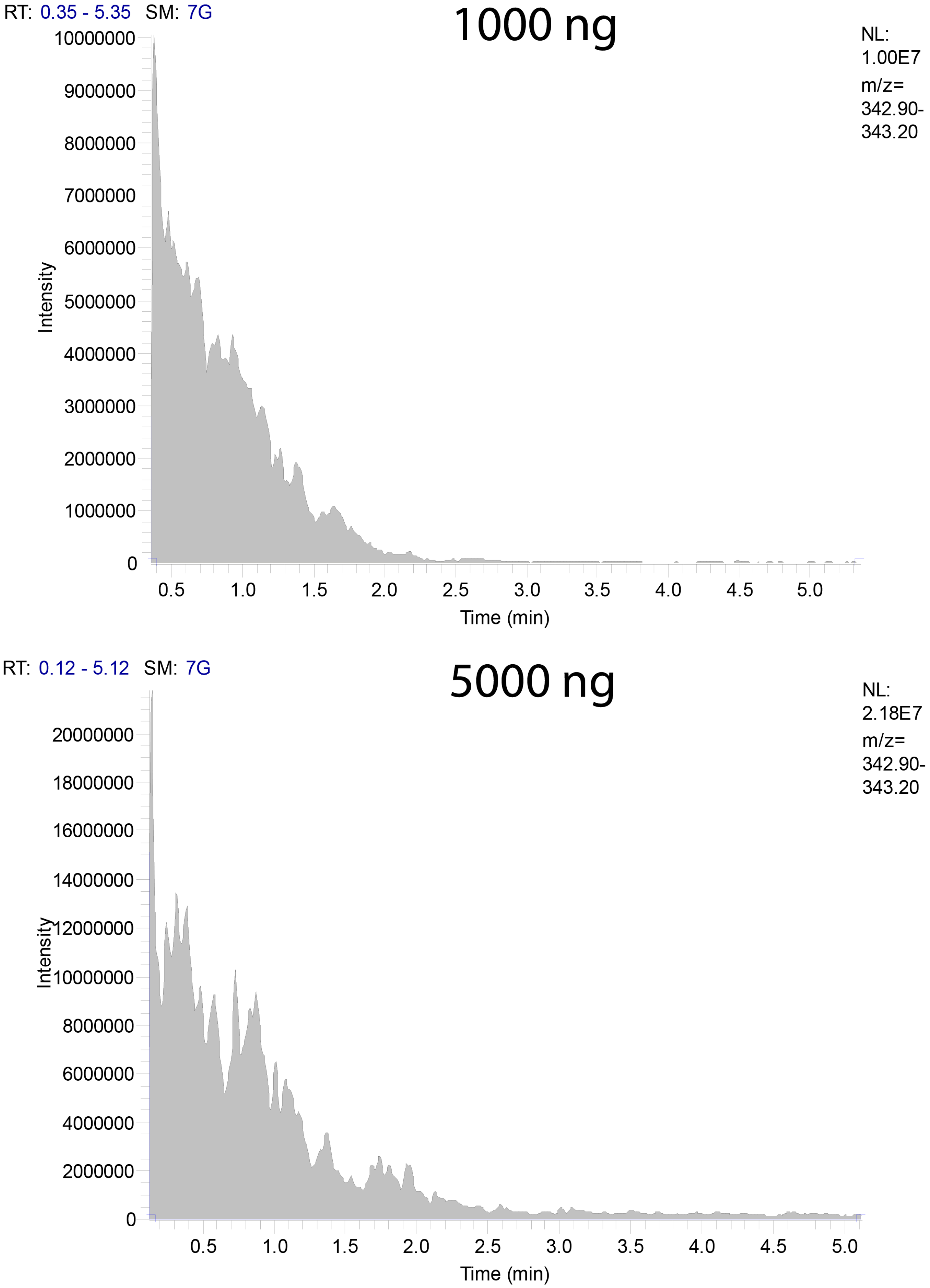

Figure S4 Depletion curves of 1000 ng and 5000 ng boscalid, obtained by an extracted ion current of m/z 342.90-343.20 (gaussian smoothing 7 points).

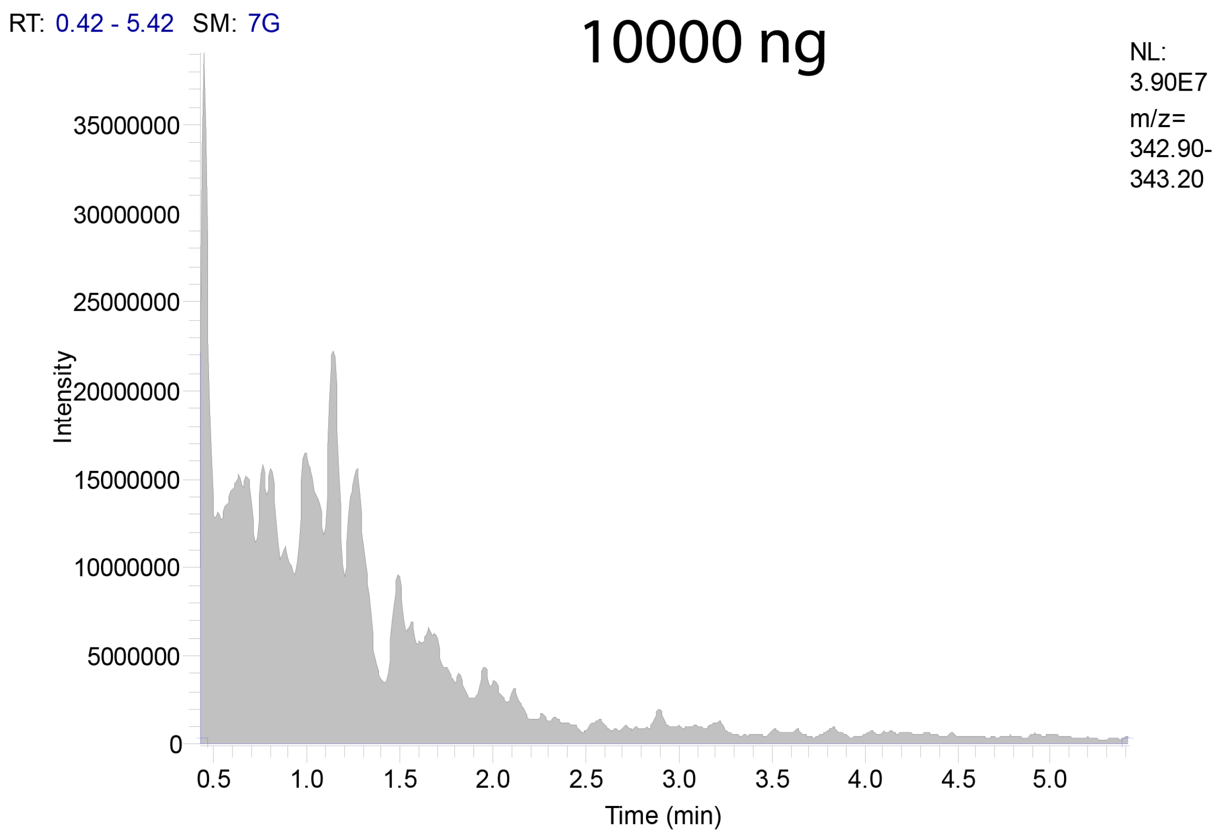

Figure S5 Depletion curve of 10000 ng boscalid, obtained by an extracted ion current of m/z 342.90-343.20 (gaussian smoothing 7 points).

S4 Detection of low pesticide amounts: a) boscalid (5 ng), b) imazalil (1 ng), and c) thiabendazole (2 ng)

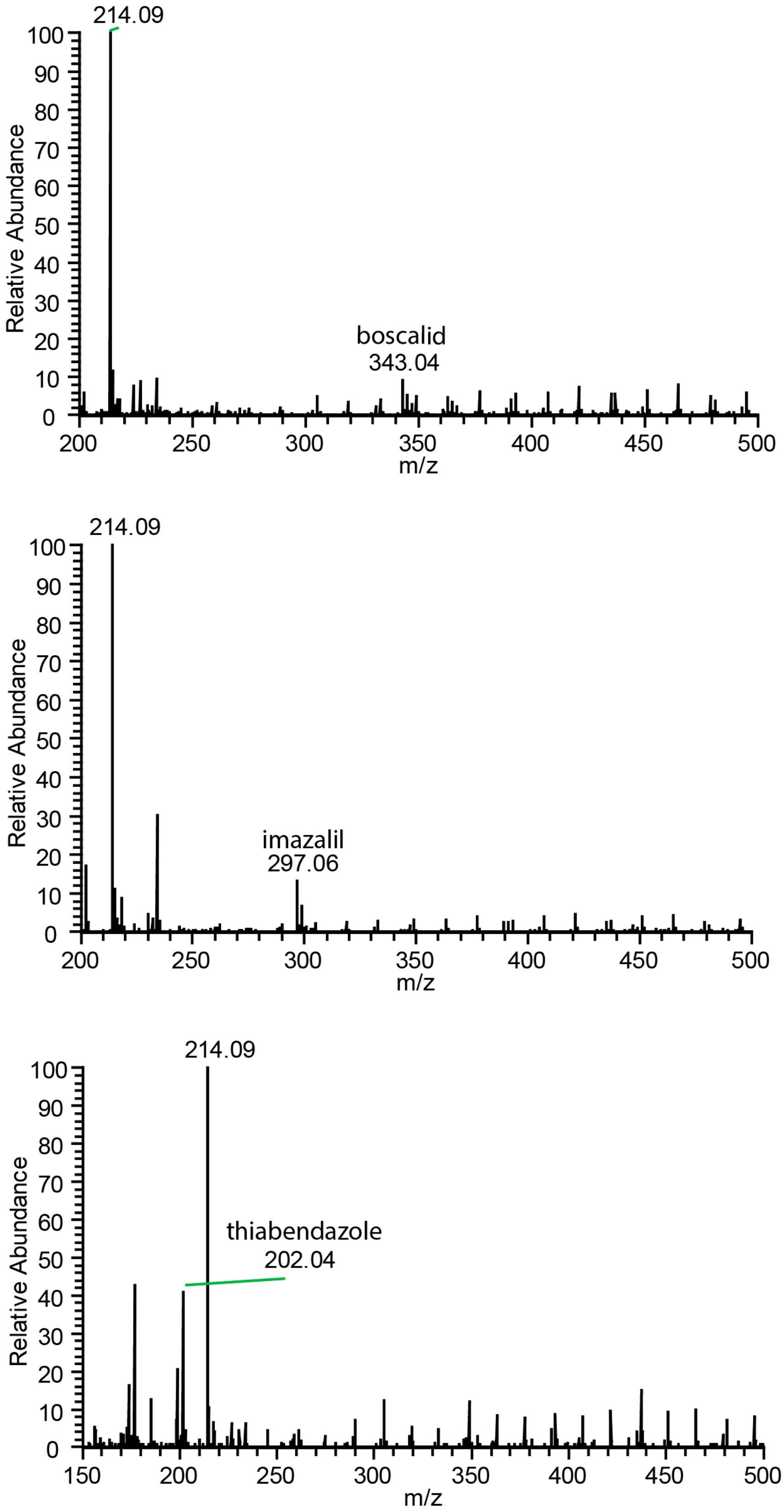

Figure S6 Full scan spectra of the protonated pesticides.

S5 Boscalid quantitation on bell pepper

Table S2 Boscalid quantitation on bell pepper.

The integrated depletion curve area of 3.47 x 10^8 counts is compared to the calibration curve:	$3.47 * 10^8 = 4 * 10^6 * x^{0.6169}$
The equation is solved for x:	x ≈ 1387 ng

This yields 1.4 µg (1387 ng) per 15 cm^2 sampling area, which amounts to 19 µg for the complete surface area of 200 cm^2. Based on the bell pepper weight of 232 g, the pesticide concentration corresponds to 82 µg per kg bell pepper.

S6 Suppression of marker

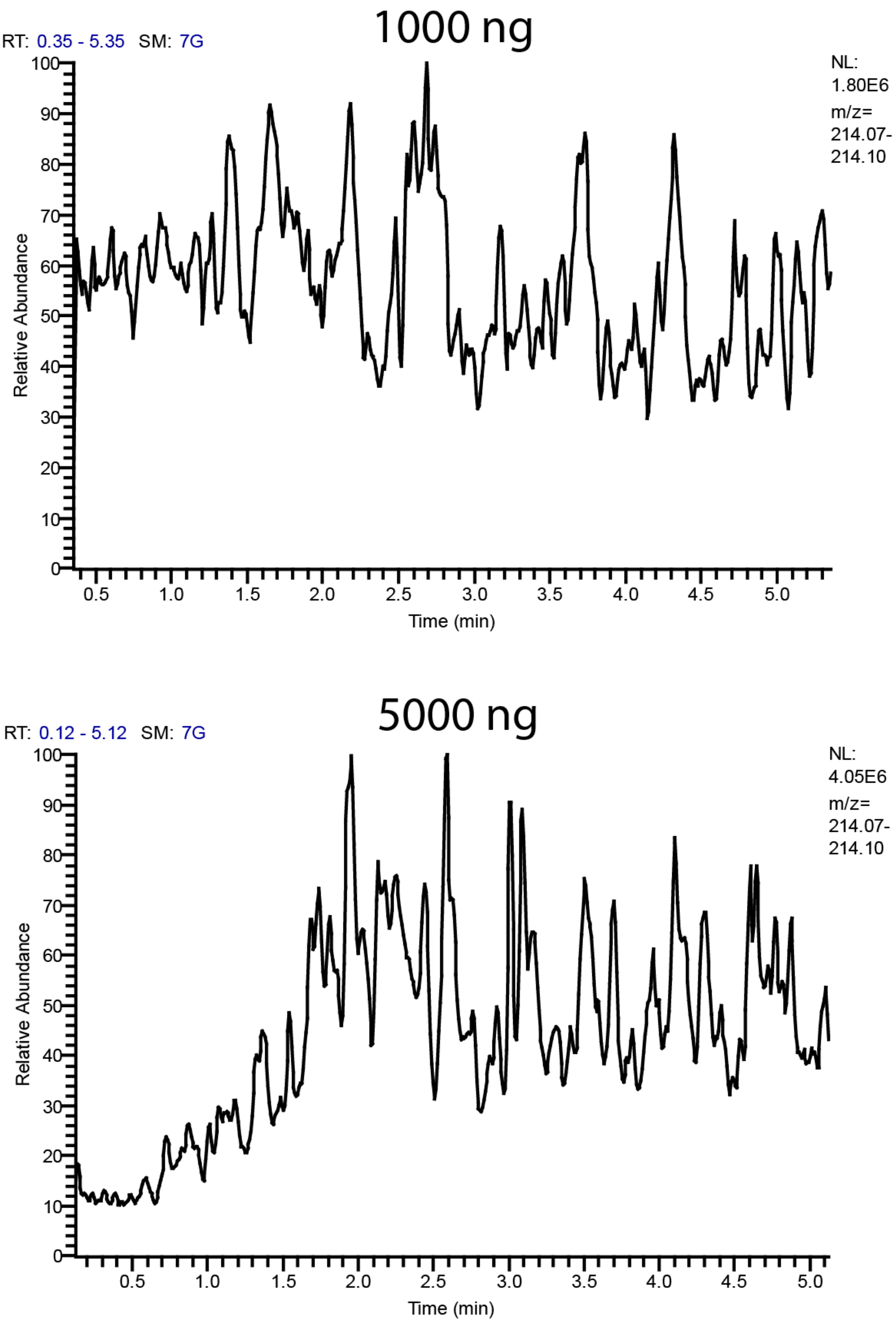

Figure S7 The extracted ion current of the suppression marker is visualized during an extraction period of five minutes after deposition of 1000 ng boscalid, shown at the top, and 5000 ng boscalid, shown at the bottom. The deposition of 5000 ng boscalid leads to suppression of the marker at the beginning of the analysis.
